# Evaluation of Individualized Functional Electrical Stimulation-Induced Acute Changes during Walking: A Case Series in Children with Cerebral Palsy

**DOI:** 10.3390/s21134452

**Published:** 2021-06-29

**Authors:** Nicole Zahradka, Ahad Behboodi, Ashwini Sansare, Samuel C. K. Lee

**Affiliations:** 1 Biomechanics and Movement Science Program, University of Delaware, Newark, DE 19713, USA; nzahrad1@jhu.edu (N.Z.); ahadbeh@udel.edu (A.B.); ashwini@udel.edu (A.S.); 2 Department of Physical Therapy, University of Delaware, Newark, DE 19713, USA; 3 Shriners Hospitals for Children, Philadelphia, PA 19140, USA

**Keywords:** functional electrical stimulation (FES), cerebral palsy, gait

## Abstract

Functional electrical stimulation (FES) walking interventions have demonstrated improvements to gait parameters; however, studies were often confined to stimulation of one or two muscle groups. Increased options such as number of muscle groups targeted, timing of stimulation delivery, and level of stimulation are needed to address subject-specific gait deviations. We aimed to demonstrate the feasibility of using a FES system with increased stimulation options during walking in children with cerebral palsy (CP). Three physical therapists designed individualized stimulation programs for six children with CP to target participant-specific gait deviations. Stimulation settings (pulse duration and current) were tuned to each participant. Participants donned our custom FES system that utilized gait phase detection to control stimulation to lower extremity muscle groups and walked on a treadmill at a self-selected speed. Motion capture data were collected during walking with and without the individualized stimulation program. Eight gait metrics and associated timing were compared between walking conditions. The prescribed participant-specific stimulation programs induced significant change towards typical gait in at least one metric for each participant with one iteration of FES-walking. FES systems with increased stimulation options have the potential to allow the physical therapist to better target the individual’s gait deviations than a one size fits all device.

## 1. Introduction

Cerebral palsy (CP) is a non-progressive disorder caused by a lesion of the fetal or infant brain that results in muscle weakness, spasticity, and other motor impairments that impact functional mobility. CP is the most prevalent childhood neuromotor diagnosis with an estimate of 764,000 people having CP and approximately 10,000 new cases in the US each year [1]. A range of 70–80% percent of the CP population is classified with spastic CP [2,3] which is characterized by clinical and functional impairments of decreased passive joint range of motion, increased muscle spasticity/tone, impaired coordination, decreased muscle strength, and diminished ability to ambulate without assistance or assistive devices [4,5,6].

The normal progression of CP is a loss of walking function over time that is characterized by spatiotemporal and kinematic deviations; most children become less independent with functional mobility throughout adolescence and adulthood [7,8,9,10,11,12]. Contributing factors to this deterioration are muscle weakness, caused by a lack of muscle strength gains proportional to stature and weight [13], and the presence of spasticity. These underlying factors manifest in deviations from typical gait [14] with the largest deviations observed in the hip, knee, and ankle angles in the sagittal plane [15,16,17]. The gait often observed in CP [14,18,19] results in inefficient walking and increased energy expenditure [20,21,22].

Functional electrical stimulation (FES) of impaired muscles is an approach to reduce gait deviations by targeting both the underlying causes of gait deviations and walking itself. Previous studies demonstrated that FES produces changes in walking towards more typical patterns [23,24]. FES-assisted walking has improved underlying impairments and functional outcomes, such as spatiotemporal parameters [25,26,27], passive range of motion [26], kinematics [28,29,30], and kinetics [30]. Specific improvements seen with FES assistance include improved ankle and knee angles, cadence, step length, and walking velocity [23,26,29,30,31]. Studies report that kinematic improvements occur based upon the muscle groups targeted; stimulation to the gastrocnemius only [24], dorsiflexors only [28,29,31,32,33], or gastrocnemius and tibialis anterior combined [24,31] promoted changes at the ankle while stimulation to the quadriceps [28,34] improved knee flexion angle, and continuous low-level stimulation of gluteus medius [35] improved hip abduction. Additionally, by changing the kinematics at one joint with FES, there is potential concomitant impact to other joints as well [36]. Assessing the effects of a FES strategy during walking in children with CP, however, has been difficult due to the heterogeneity of the study population [37,38].

A one size FES program does not fit all individuals with CP as improvements are influenced by the variation of gait patterns [39] and individual responses to FES [28,29]. To address the variability in gait deviations, it is necessary to have custom tailored stimulation programs. Additional features and options within the FES system are needed in currently available commercial systems when used in walking interventions such as the ability to target more muscle groups in different combinations, and an increased control over the timing of stimulation delivery. The Odstock Dropped Foot Stimulator systems (Biomedical Engineering and Medical Physics, Salisbury, UK), Respond II Select (Medtronic Inc., Minneapolis, MN, USA), and Ness L300^®^ Plus (Bioness Inc., Valencia, CA, USA) are systems capable of stimulating one or two muscle groups. The RehaMove gait training application (Hasomed Inc., Magdeburg, Germany) is capable of stimulating eight channels but uses feedforward sequencing with limited stimulation timing control. There is a lack of available FES systems that have both sophisticated control, such as finite-state control with high resolution, and address more than two muscle groups during walking.

Targeting multiple joints with stimulation to multiple muscle groups has an additive effect on gait improvements [40] compared to varying results found in studies that target a single joint with stimulation to multiple muscle groups [31,41,42]. For example, stimulation of both rectus femoris and vastus lateralis were more effective in improving posture than stimulation of each muscle alone during walking in one child with CP [43]. Behboodi et al. demonstrated alterations toward typical gait in hip and ankle angles after 12 weeks of training with subject-specific FES programs targeting multiple muscle groups in two individuals [44]. Similar results were shown by Rose et al. in three participants using a multi-channel FES system [45]. Although case studies have recently demonstrated the potential of improving gait when targeting multiple joints with multi-channel FES systems [44,45], evidence is limited [40]. Advancement in FES technology resulting in improved stimulation timing may contribute to differences between older and more recent multi-channel FES studies [40]. More importantly, older FES walking studies lacked subject-specific FES programs that limited the flexibility of an intervention needed for a heterogeneous population with heterogeneous gait deviations.

A FES program must be able to target multiple joints by stimulating multiple muscle groups at the correct times in the gait cycle and account for the asymmetrical gait deviations in the CP population [46]. The purpose of this case series is to demonstrate the feasibility of prescribing subject-specific stimulation programs for children with CP using a flexible FES system to target their subject-specific gait deviations.

## 2. Materials and Methods

### 2.1. Functional Electrical Stimulation

#### 2.1.1. System

A custom FES system was designed with increased gait phase detection capability [47] and flexibility to target up to 12 channels with stimulation during walking. The multichannel surface FES system incorporated two inertial measurement units (IMUs) (Opal^TM^, APDM, Portland, OR, USA), two stimulators (RehaStim, Hasomed Inc., Magdeburg, Germany), and a custom gait phase detection (GPD) algorithm (LabVIEW, National Instruments, Austin, TX, USA) [47,48] to provide finite-state control over the timing of stimulation delivery. Predefined rules were established to distinguish between the seven phases of gait—loading response (*LR*), mid-stance (*MSt*), terminal stance (*TSt*), pre-swing (*PSw*), initial swing (*ISw*), mid-swing (*MSw*), and terminal swing (*TSw*)—derived from shank angular velocity. The GPD system was validated during walking in children with cerebral palsy; onset detection of gait phases determined by the GPD algorithm had root mean square errors that ranged from 63 ms (*LR and PSw*) to 127 ms (*MSw*) when compared to motion capture [48].

A FES trigger compensation strategy accounted for the inherent delays of the GPD system [49]. A trigger was created when 75% of the current phase duration was reached to stimulate muscle groups associated with the next gait phase. The time associated with percentage delay was gait phase dependent and equal to a percentage of the average duration of the gait phase. This pre-trigger strategy was piloted in an adolescent with cerebral palsy (CP) (16-year-old, male) and resulted in an average stimulation onset difference of 1.5% gait cycle (GC) and range of 7% GC; stimulation signals occurred 5% GC earlier and 2% GC later than the desired time [49].

By incorporating the gait phase detection system, designed specifically for the CP population, some of the stimulator settings increased in customization flexibility. For example, stimulation pulse duration was customizable for each of the seven gait phases. A custom-designed LabVIEW-based application programming interface (API) was capable of updating the stimulation protocol, including stimulation delivery timing, stimulation intensity (current and pulse duration), and the target muscles (stimulation channels) in real-time, while subjects walked on a treadmill. Note that the RehaStim stimulators that were integrated into our FES system used feedforward control for their gait-related applications.

#### 2.1.2. Stimulation Programming

Stimulation programming included (1) targeted lower extremity muscle group(s), (2) gait phase(s) to initiate and terminate stimulation, (3) stimulation current for each muscle group, and (4) stimulation pulse duration (intensity) for each muscle group and gait phase. The lower extremity muscle groups to select from were the gluteals, hamstrings, quadriceps, dorsiflexors, and/or plantarflexors. Right and left sides were programmed independently. Gait phase onset to select from were *LR*, *MSt*, *TSt*, *PSw*, *ISw*, *MSw*, and *TSw* to control stimulation initiation and termination. Stimulation current and pulse duration ranges were 20–130 milliamps (mA) and 20–500 microseconds (µs), respectively. Stimulation frequency was fixed at 40 Hz and a charge-balanced bi-phasic waveform was used for stimulation.

Stimulation current and pulse duration values were set to elicit a functional muscle response (concentric muscle contraction) for each muscle group and determined by a stimulation thresholding process (below). Fifty percent of the pulse duration value for a functional response was set as the pulse duration for an eccentric contraction of the targeted muscle group to avoid restricting limb advancement to the next gait phase. A standardized reduction in pulse duration was used to maintain consistency when setting the pulse duration for reduced level stimulation while supporting individualized pulse durations to produce functional responses.

#### 2.1.3. Stimulation Thresholding

Stimulation thresholding was a two-step process to determine stimulation parameter values (current and pulse duration) associated with a functional response. Stimulation parameter values from step one (baseline) were refined in step two (fine-tuned) and programmed into the stimulation algorithm.

For baseline, stimulation parameter values were determined to produce a motor response. The participant stood in a neutral position with weight distributed equally between both feet and stimulation parameter values were determined for each muscle group. Initial stimulation current was set to 30 mA and pulse duration was ramped up until a motor response was observed. If pulse duration reached 250 µs without an observed response, current was increased by 10 mA and pulse duration was reevaluated. This was iterated upon until a combination of a stimulation current and a pulse duration less than 250 µs produced a motor response. The current was raised before maximum pulse duration was reached to have a pulse duration range available during fine-tuning.

For fine-tuning, stimulation parameter values were determined to produce a specified functional movement associated with the muscle targeted with stimulation (Figure S1). The participant’s limb was positioned to mimic its position during the stance or swing period of gait. A secondary position was used to represent specific gait phases—i.e., the foot was positioned to mimic placement during pre-swing. The position for the stance period was defined as standing in a neutral position unless including a secondary position. The position for the swing period was defined as single leg standing and required stability on the leg that was not being evaluated while the leg of interest was non-weight bearing. If the participant was not capable of maintaining balance during single leg stance, fine-tuning was completed in a lateral recumbent position. Initial stimulation parameters were set to the values obtained during baseline. The pulse duration was increased, in small increments (~5 µs), until functional movement was achieved as determined by the physical therapist’s clinical judgement. If 500 µs was reached before producing the functional movement, the current was increased by 10 mA and the pulse duration level was reevaluated. This was iterated upon similar to baseline until the functional movement was achieved or the participant’s maximum tolerance was reached. If the participant was not able to tolerate stimulation to the muscle group, the muscle group was excluded from the stimulation program.

### 2.2. Experimental Protocol

Six children with CP (4 Male, 14 ± 2 years old) participated in the FES walking study. Participants were recruited through an outpatient CP clinic, local referral sources, and Institutional Review Board (IRB) approved advertisements and recruitment flyers. Parental permission and child assent were obtained from eligible participants (Table S1) prior to participation.

#### 2.2.1. Muscle Selection and Timing of Stimulation Delivery

Frontal and sagittal videos of the individual walking for at least 10 consecutive steps were visually inspected by three physical therapists to identify gait deviations. The physical therapists were experienced in identifying gait deviations in CP and convened a post-evaluation to compare their separate observations. Consensus was achieved through discussion when evaluator opinions differed on the targeted muscle group, timing, and/or level of stimulation (concentric vs. eccentric contraction) to improve gait.

#### 2.2.2. Data Collection

Before the data collection, the participant’s height and weight were collected and self-selected over-ground walking speed was derived from the 10-m walk test (MWT) [50]. A comfortable speed was established, based on participant feedback, if self-selected over-ground speed was too fast on the treadmill.

Setup for stimulation delivery included electrode placement based on Robinson and Snyder-Mackler placement protocol [51] on muscles targeted by the individual’s stimulation program. A low level of stimulation was delivered to ensure appropriate electrode placement and stimulation thresholding followed. If stimulation thresholding occurred on the day before gait analysis, markings were made on the legs to identify the corners of each electrode and served as a guide for placement the next day. Stimulation intensities were retested.

Participants donned the FES system while walking at a self-selected treadmill speed on the split-belt treadmill (Bertec, Columbus, OH, USA). Orthotics and assistive devices were not permitted but the side handrails on the treadmill were available for support if needed. A non-weight bearing harness was used for safety in the event of stumbles or falls. Kinematic and kinetic data were captured using an eight-camera motion capture system (Motion Analysis Corporation, Santa Rosa, CA, USA) with a sampling rate of 128 Hz and two force places (Bertec, Columbus, OH, USA) with a sampling rate of 3200 Hz, respectively. A modified Cleveland Clinic marker set was used and data were collected for three walking conditions: *noFES1*, *FES*, *noFES2*. After a treadmill-walking accommodation period [52], a 30-s walking trial was collected without stimulation (*noFES1*). The individual’s stimulation program was applied while the participant walked at a comfortable speed on the treadmill (*FES*) and data were collected following a 30-s period to acclimate to the stimulation. A walking trial without stimulation was collected at the end (*noFES2*). Each condition was separated by five minutes of seated rest. The walking conditions were part of a larger randomized data collection session where multiple stimulation programs were tested.

#### 2.2.3. Typically Developing (TD) Reference Dataset

A reference dataset was generated from gait analysis of seven typically developing (*TD*) children (5 Females, 12.4 ± 2.15 years old). Participants were locally recruited and IRB approved parental consent and child assent documents were obtained prior to participation. Kinematic and kinetic data were collected for a 30-s walking trial while individuals walked on an instrumented treadmill at a self-selected treadmill speed. Self-selected speed was derived from the 10 MWT and treadmill speed was adjusted from this speed, if needed, based on participant feedback. Kinematic and kinetic data were captured using the same motion capture setup and protocol as participants with CP.

### 2.3. Gait Analysis

Marker and force plate data were processed retrospectively in Visual 3D (C-Motion Inc., Germantown, MD, USA) and filtered using a Butterworth low pass filter with cutoff frequencies of 6 and 25 Hz, respectively. A threshold of 20 N was applied to the ground reaction force (GRF) to determine the timing of initial and terminal contact [53]. These gait events, initial contact (*IC*) and terminal contact (*TC*), were visually inspected for accuracy. Instances of incorrect assignment of *IC* or *TC* resulting from atypical gait such as ‘toe-dragging’ were corrected by deleting the event. The number of missed events determined how many gait cycles were included from the last 10 consecutive gait cycles on each side. Kinematic and kinetic data were normalized to the participant’s anthropometric measures, such as height and weight, and joint angles (hip, knee, and ankle) were calculated relative to the proximal segment with a right-handed Cardan X-Y-Z (mediolateral, anteroposterior, vertical) rotation sequence in Visual 3D. The last 10 consecutive right and left gait cycles from the three walking conditions in CP (*noFES1*, *FES*, *noFES2*) and walking in typically developing children (*TD*) were included in gait metric calculations. Gait metrics include peak propulsive force (*AGRF*), minimum toe clearance in swing period (*TC_sw*), maximum hip extension angle (*HME*), maximum knee extension angle in swing period (*KME_sw*), maximum knee extension angle in stance period (*KME_st*), maximum knee flexion angle in stance period (*KMF_st*), maximum hip external rotation in stance period (*HMER_st*), and maximum hip external rotation in swing period (*HMER_sw*) (Table 1). Right *HMER_st* and *HMER_sw* values were normalized to the left side (negated) for summary metrics. Absolute time associated with a gait metric was extracted for AGRF (*t_AGRF_*), TC_sw (*t_TC_sw_*), HME (*t_HME_*), KME_sw (*t_KME_sw_*), KME_st (*t_KME_st_*), KMF_st (*t_KMF_st_*), HMER_st (*t_HMER_st_*), and HMER_sw (*t_HMER_sw_*) and expressed as a percentage of gait cycle. Time normalization to percent of gait cycle was calculated using a custom program in MATLAB (The MathWorks, Natick, MA, USA). Note that for each participant with CP, only the gait metrics and timing of gait metrics associated with the target gait deviations identified by the physical therapists were calculated.

### 2.4. Statistics Analysis

Gait metrics (*AGRF*, *TC_sw*, *HME*, *KME_sw*, *KME_st*, *KMF_st*, *HMER_st*, *HMER_sw*) and timing of gait metrics (*t_AGRF_*, *t_TC_sw_*, *t_HME_*, *t_KME_sw_*, *t_KME_st_*, *t_KMF_st_*, *t_HMER_st_*, *t_HMER_sw_*) were summarized using median and interquartile range (IQR). Gait metrics and timing of gait metrics were plotted with 95% confidence intervals (CI) of the median. Three comparisons were made: *noFES1* vs. *TD, noFES1* vs. *FES, noFES1* vs. *noFES2.* Mann–Whitney U tests of gait metrics were used to assess *noFES1* vs. *TD* to validate physical therapist’s determination of gait deviations. Wilcoxon signed rank tests of gait metrics and timing of gait metrics were used to compare walking conditions for each participant with CP: *noFES1* vs. *FES*, *noFES1* vs. *noFES2*. *p* values with Bonferroni correction were applied in multiple comparison analysis tests to identify: (1) gait metrics that were significant for *noFES1* vs. *noFES2*, (2) gait metrics that were significant for *noFES1* vs. *FES*, (3) timing of gait metrics that were significant for *noFES1* vs. *noFES2*, and (4) timing of gait metrics that were significant for *noFES1* vs. *FES* (*p* < 0.05). All statistical analyses were performed using GraphPad Prism 9 Version 9.1.0. (GraphPad Software, San Diego, CA, USA).

## 3. Results

Six children with CP participated in the FES-assisted walking study. Functional mobility level of the participants was either Gross Motor Function Classification System (GMFCS) level II or III (Table 2) [56]; different types of assistive devices were used by participants classified as GMFCS III. GMFCS II is characterized as the ability to walk in most settings but experiencing difficulty with longer distances and GMFCS III is characterized as the use of hand-held mobility devices in most indoor settings with use of wheeled mobility when traveling long distances [57]. Self-selected walking speeds ranged from 0.60–1.07 m/s and all participants used a decreased walking speed on the treadmill (Table 2).

### 3.1. Individualized Stimulation Programs

**Figure 1:**
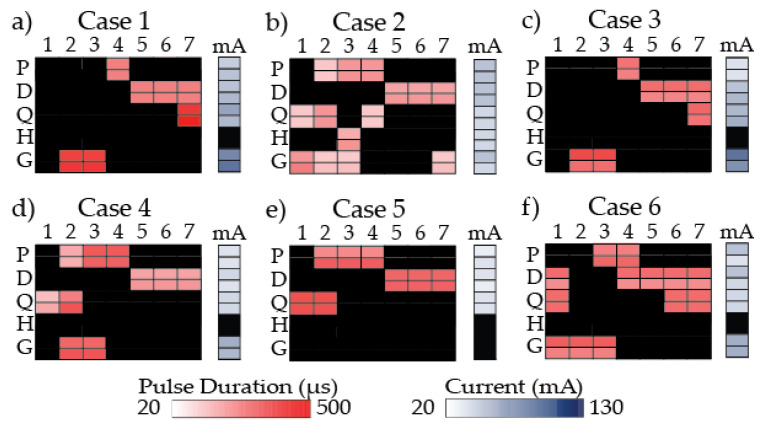
Stimulation program for each participant. A heat map shows the pulse duration (µs) used for each gait phase (column) and muscle group (row) and current (*mA*) used for each muscle group. For each muscle group, there is a left side (top row) and right side (bottom row). Darker colors are associated with higher pulse duration in red and higher current in blue. Black indicates no stimulation. 1: loading response; 2: mid-stance; 3: terminal stance; 4: pre-swing; 5: initial swing; 6: mid-swing; 7: terminal swing; mA: current; P: plantarflexors; D: dorsiflexors; Q: quadriceps; H: hamstrings; G: gluteals.

Stimulation programs were designed by three physical therapists to target gait deviations identified in frontal and sagittal walking videos for each participant (Figure 1).

Case 1 stimulation program was created to increase push-off power, toe clearance in swing period, hip and knee extension in stance period, and help progress the leg forward. However, the stimulation program was adjusted during the data collection because the participant demonstrated inadequate forward progression of the leg during stance period when the initial stimulation program was applied. Modifications to improve forward progression included stimulation to plantarflexors during *PSw* only instead of *MSt* through *PSw*, excluding the hamstrings, and activating the quadriceps during *TSw* instead of *LR* through *MSt* (Figure 1a).

Case 2 stimulation program was created to increase push-off power, toe clearance in swing period, hip external rotation in swing period, knee extension in stance period, knee flexion at the end of stance period, and to progress the leg forward at the end of stance period. A secondary strategy to target the quadriceps from *PSw* to *ISw* to aid in leg progression at the end of stance period was tested and used (Figure 1b).

Case 3 stimulation program was created to increase push-off power, toe clearance in swing period, hip and knee extension in stance period, stability in stance period, and leg extension (*KME_sw*) at the end of swing period. Modifications included targeting quadriceps during *TSw* only because the participant showed adverse effects to stimulation of the quadriceps in stance period; his legs locked in extension. The plantarflexors were modified to *PSw* only (Figure 1c).

Case 4 stimulation program was created to increase push-off power, toe clearance in swing period, hip external rotation in stance period, and knee extension in stance period (Figure 1d).

Case 5 stimulation program was created to increase push-off power, toe clearance in swing period, stability during weight bearing, and hip and knee extension in stance period. Targeting the gluteals from *LR* to *TSt* was removed from the stimulation program because the participant was unable to tolerate stimulation to the gluteal muscles (Figure 1e).

Case 6 stimulation program was created to increase push-off power, toe clearance in swing period, hip extension in stance period, and knee extension in swing period, and to pre-position the leg better for weight bearing (Figure 1f).

### 3.2. Typically Developing (TD) Reference Dataset

Gait analysis was performed on seven typically developing children to generate a reference dataset. One-hundred and forty gait cycles were included in the TD gait metrics and timing of gait metrics (Table 3).

### 3.3. Gait Metrics and Timing in CP

Gait analysis was performed on six children with cerebral palsy; median of gait metrics and timing of gait metrics targeted by the participant’s stimulation program were calculated for *noFES1*, *FES*, and *noFES2* walking conditions. *AGRF*, *t_AGRF_*, *TC_sw*, and *t_TC_sw_* were calculated for all cases. *HME* and *t_HME_* were calculated for Case 1, 3, 5, and 6. *KME_sw* and *t_KME_sw_* were calculated for Case 3 and 6 while *KME_st* and *t_KME_st_* were calculated for all cases except Case 6. *KMF_st*, *t_KMF_st_*, *HMER_sw*, and *t_HMER_sw_* were calculated for Case 2 and *HMER_st* and *t_HMER_st_* were calculated for Case 4. Note that for each case only the gait metrics associated with gait deviations targeted by the participant-specific stimulation programs, which were prescribed by the physical therapists, were calculated. Gait metrics and timing of gait metrics included 20 gait cycles except for the condition/gait metric that had missing gait events. Nineteen gait cycles were included in the gait metric and timing of gait metric medians in *noFES1* for Case 3 (*AGRF*, *t_AGRF_*); *FES* for Case 1 (*HME*, *t_HME_*, *KME_st*, *t_KME_st_*), Case 2 (*HMER_sw*, *t_HMER_sw_*), and Case 6 (*KME_sw*, *t_KME_sw_*); and *noFES2* for Case 2 (*HMER_sw*, *t_HMER_sw_*) and Case 6 (*t_AGRF_*, *KME*_sw, *t_KME_sw_*). Ten left gait cycles in *noFES2* for Case 5 were analyzed for *TC_sw* and *t_TC_sw_* because the right toe marker fell off during the trial.

Gait metrics targeted by participant’s stimulation program were compared between *noFES1* vs. *TD* to validate the physical therapist’s determination of gait deviations. Gait metrics were significantly different (*p* < 0.05) between the *noFES1* walking condition and the typically developing reference dataset for all cases (Table 4).

**Figure 2:**
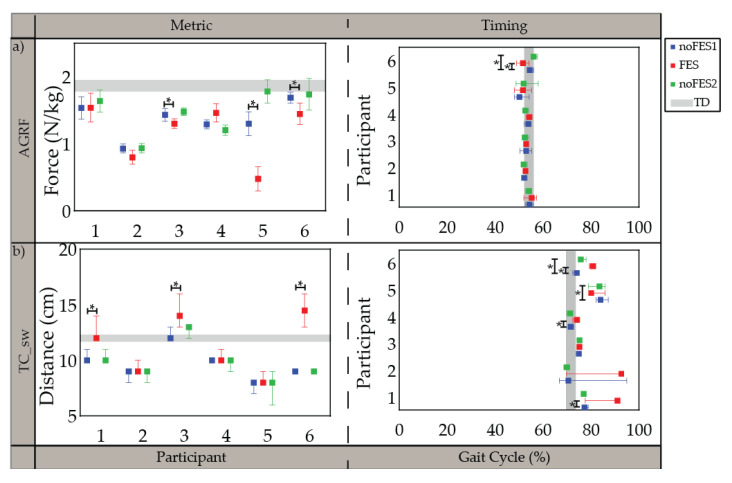
Median and 95% confidence interval (CI) of gait metrics targeted by all individualized stimulation programs and timing of gait metrics (percent of gait cycle) during *noFES1* (blue)*, FES* (red)*,* and *noFES2* (green) walking conditions. (**a**) peak propulsive force (*AGRF*) and (**b**) minimum toe clearance in swing period (*TC_sw*). Gray band represents 95% CI for typically developing reference dataset (*TD*). * indicates significance between walking conditions.

**Figure 3:**
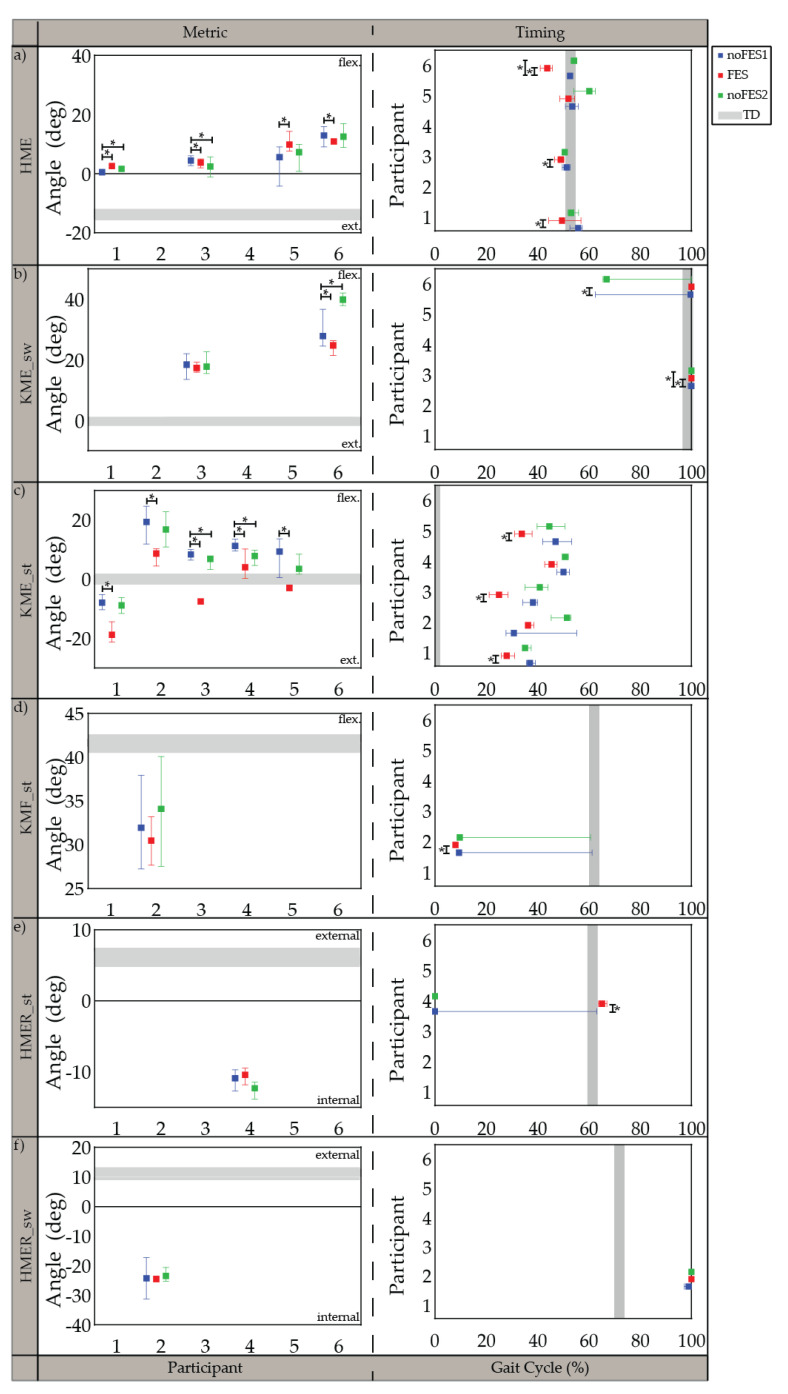
Median and 95% confidence interval (CI) of gait metrics targeted by the individualized stimulation program and timing of gait metrics (percent of gait cycle) during *noFES1* (blue)*, FES* (red)*,* and *noFES2* (green) walking conditions. Gait metrics were excluded if they were not targeted in the individual’s stimulation program. (**a**) maximum hip extension angle (*HME*), (**b**) maximum knee extension angle in swing period (*KME_sw*), (**c**) maximum knee extension angle in stance period (*KME_st*), (**d**) maximum knee flexion angle in stance period (*KMF_st*), (**e**) maximum hip external rotation in stance period (*HMER_st*), (**f**) maximum hip external rotation in swing period (*HMER_sw*). Gray band represents 95% CI for typically developing reference dataset (*TD*). * indicates significance between walking conditions.

All participants were able to walk with the FES system and tolerate stimulation with exception of the specific muscles indicated in the individualized simulation programs section (Section 3.1). The participants’ movements initially appeared robotic but over a brief acclimation period of 30 s became more natural. Gait metrics and timing of gait metrics varied in significance between participants when walking (1) without FES assistance compared to walking with FES assistance (*noFES1* vs. *FES*) and (2) without FES assistance between the first and second *noFES* conditions (*noFES1* vs. *noFES2*) (Figure 2 and Figure 3). In Figure 2 and Figure 3, gait metrics and timing of gait metrics of the participants with CP are more typical when they are closer to gray band. The gray band represents the typical range of gait metrics and timing of gait metrics and were derived from the *TD* dataset. Improvements in gait metrics and associated timing were improved when they changed in the direction of the typical range. *TC_*sw significantly improved and was in the typical range during *FES* compared to the *noFES* conditions in Case 1 (Figure 2b). Case 6 significantly improved *TC_sw* (Figure 2b), *HME* (Figure 3a), and *t_KME_sw_* during *FES* (Figure 3b). Similarly, *FES* improved *KME_st* in Cases 2 and 5 and *t_KME_st_* in Cases 1, 3, and 5 (Figure 3c). Timing of *KME_st* and *HMER_st* significantly improved during *FES* in Case 2 (Figure 3d) and Case 4 (Figure 3e), respectively.

## 4. Discussion

The feasibility of using a FES system with individualized stimulation programs during walking was evaluated in six children with CP. Stimulation programs were designed to target participant-specific gait deviations. Physical therapists used clinical judgement to identify gait deviations and the lower extremity muscles to target/timing of stimulation delivery to improve gait. Gait metrics between *noFES1* and *TD* conditions were compared to validate the physical therapists’ decisions (Table 4). Gait metrics were significantly different between the two conditions for all gait metrics targeted by the participant’s stimulation program confirming that the physical therapists were capable of targeting participants’ gait deviations via prescribing the stimulation patterns.

The variation in lower extremity muscle groups targeted and timing of stimulation delivery of the individualized stimulation programs demonstrated the utility of a flexible FES system (Figure 1). Physical therapists chose proximal muscle groups (gluteals, hamstrings, and/or quadriceps) in addition to distal muscle groups (plantarflexors and/or dorsiflexors) to correct gait deviations when given the option to target multiple muscle groups with stimulation. The flexibility of programming the stimulation algorithm “on the fly” allowed the physical therapists to make secondary strategy recommendations to be tested and integrated (Case 2). It also enabled modifications to the stimulation program to account for participant reactions to stimulation (Case 1, 3, 5). Adjustments were based on visual observation of the immediate responses to FES and the research physical therapist’s clinical judgement.

Based on typically developing muscle activity patterns during gait, targeting proximal muscle groups with stimulation at appropriate times in the gait cycle requires a higher timing resolution than detection of stance and swing periods [18]. Our FES system’s ability to use detection of seven gait phases as a trigger increased the timing control of the delivery of stimulation and provided therapists with the option to turn stimulation on/off during different phases of the swing period. FES systems in the literature were not capable of distinguishing the different gait phases that occur during swing; limiting control of stimulation during this period. In previous studies which targeted only the plantarflexors and/or dorsiflexors [27,32,57], gait phase resolution beyond stance and swing periods was not needed to achieve sufficient stimulation delivery control. Individualized stimulation programs that included gluteals and quadriceps were programed to trigger stimulation later in swing period during *MSw* or *TSw*; increased gait phase resolution was essential.

Stimulation current and pulse duration were programmable for each muscle group and muscle group/gait phase, respectively. The level of stimulation needed to create a functional movement was able to be tailored to each muscle group and gait phase. It also enabled different levels of stimulation to the same muscle at different gait phases to create a functional movement during some phases and to assist in controlling the limb without restricting the movement during others. For example, in both Cases 2 and 4, a functional level of stimulation to the plantarflexors was used during *TSt* and *PSw* to promote push-off power (concentric muscle activation) while a reduced level of stimulation was used during *MSt* to assist in controlling the progression of the shank over the foot (eccentric muscle activation) without restricting the motion. Only two levels of stimulation for each muscle group were used for this study. For each muscle group, however, the FES system supported the programming of different pulse durations for each phase of gait. The increased options of the FES system gave the physical therapists the opportunity to adjust the stimulation programs based on the individual’s gait deviations as well as the participant’s response to FES.

Walking trials without FES (*noFES1* and *noFES2*) were collected before and after walking with FES (*FES*) to isolate changes in gait metrics and timing of gait metrics resulting from FES versus confounding factors such as learning or time [58]. Gait metrics and timing of gait metrics that were significantly different between *noFES1* and *noFES2* indicated that significance of these variables found between *noFES1* and *FES* was inconclusive as to the cause of change.

The FES system was deployed as a wearable device during walking in six children with CP. Seven phases of gait were detectable in all cases [48] and used to control stimulation delivery in combination with the FES trigger compensation strategy. Participants demonstrated changes in individual gait metrics associated with one iteration of participant-specific stimulation prescription for FES walking. Push-off power (*AGRF*) and toe clearance in swing period (*TC_sw*) were gait metrics targeted by the stimulation program in all six cases (Figure 2). Different strategies (muscle groups/timing of stimulation delivery) were used between different cases to target the same gait metric improvements suggesting that tailoring the stimulation program to the individual is necessary to account for the differences in gait deviations and participant responses that cannot be addressed with a one size fits all device.

Five out of six individualized stimulation programs targeted knee extension in stance period (*KME_st*). All five cases demonstrated increased knee extension with FES and were significantly different from *noFES1*. While previous studies showed increased knee extension when targeting the quadriceps with FES during walking [28,34], the stimulation program for Case 3 illustrates that improvements in knee extension during stance period are achievable without quadriceps stimulation; further illustrating that different strategies can produce similar gait metric improvements. Timing of maximum knee extension in stance period (*t_KME_st_*) was also significantly improved in three out of five cases with individualized stimulation programs.

The timing of minimum toe clearance (*t_TC_sw_*) in TD occurred during *MSw* [55], however, in participants with CP it typically occurs later in the gait cycle. The difference of *t_TC_sw_* between CP and TD may be due to the common gait abnormality in CP gait: absence of heel strike [59]. Improvement in *t_TC_sw_* during *FES* suggests that stimulation prepositioned the foot so that heel strike was present at initial contact. This improvement was similar to outcomes reported by Pool et al. [33] and Prosser et al. [60] on the effects of using the Walk Aide FES stimulator during activities of daily living (ADL) on 32 and 21 participants with CP, respectively. Both studies showed statistically significant improvements in ankle angle at *IC* and peak ankle dorsiflexion angle in swing period. Improvement in ankle dorsiflexion is one the most consistent outcomes in FES walking studies.

Although significant gait metric and timing improvements were observed in each participant, a limitation of this study was that only one iteration of stimulation settings was tested. Different stimulation parameters have the potential to resolve ‘overshooting’ the targeted gait metrics (*TD*). The timing of the delivery of stimulation was a factor that may have contributed to reduced improvements in gait metrics. The current FES compensation strategy utilized a fixed delay; however, the sophistication of the system allows for future customization of variable phase delays. FES-assisted walking was an unfamiliar task and the participants received a limited amount of practice. It is highly conceivable that with additional practice and recursive adjustments to the stimulation strategies employed, greater corrective changes throughout gait can be obtained. Nevertheless, this case series demonstrates the FES system’s flexibility for such iterations to be feasible. This case series is part of a larger FES walking intervention study where longer acclimation periods and repeat sessions to fine tune the FES has shown changes toward typical gait [44].

FES systems with increased stimulation options have the potential to allow the physical therapist to better target the individual’s gait deviations than a one size fits all device. This case series demonstrates that it is feasible to use our FES system and prescribe subject-specific stimulation programs. Although acute improvements varied between participants, each participant showed acute improvements in at least one gait metric/timing. Future FES-assisted walking intervention studies should investigate different combinations of targeted muscle groups and timing of stimulation delivery to determine the optimal program for improving walking function in each individual. To determine generalized effectiveness of the proposed FES protocol in children with CP, especially significant improvements in *KME_st and t_KME_st_*, an increased sample size is needed for each outcome measure in future studies. Due to the heterogeneity of this population, however, this may require recruitment of a high number of participants.

## Figures and Tables

**Table 1:** Definition of gait metrics.

Gait Metric	Definition
*AGRF*	Global maxima of the GRF in the anterior direction between contralateral *IC* and ipsilateral *TC* [54]
*TC_sw*	Global minima of the target path of the distal toe marker in the vertical direction between *TC* and *IC* [55]
*HME*	Global minima of hip angle during the gait cycle
*KME_sw*	Global maxima of knee angle between *TC* and *IC*
*KME_st*	Global maxima of knee angle between *IC* and *TC*
*KMF_st*	Global minima of knee angle between *IC* and *TC*
*HMER_st*	Global minima (right side) and maxima (left side) of hip rotation between *IC* and *TC*
*HMER_sw*	Global minima (right side) and maxima (left side) of hip rotation between *TC* and *IC*

**Table 2:** Characteristics of participants. (*B*) bilateral use.

Case	1	2	3	4	5	6
Sex	M	M	M	M	F	F
GMFCS ^1^	III	III	II	II	III	II
Height (m)	1.67	1.70	1.70	1.52	1.31	1.44
Weight (kg)	32.13	60.06	61.97	42.60	31.60	42.53
Self-selected speed (m/s)	0.77	0.83	0.98	1.07	0.60	0.90
Treadmill speed (m/s)	0.60	0.80	0.90	0.75	0.45	0.80
Assistive Device	(*B*) lofstrand crutches	(*B*) lofstrand crutches	-	-	Posterior and Anterior Walkers	-
Braces	-	(*B*) AFO ^2^	-	-	-	-

^1^ Gross Motor Function Classification System [56], ^2^ Ankle Foot Orthosis.

**Table 3:** TD gait metrics and timing of gait metrics.

Gait Metric.	Metric Median (IQR)	Timing (% Gait Cycle) Median (IQR)
*AGRF* (N/kg)	1.88 (0.66)	54.11 (2.83)
*TC_sw* (cm)	12.00 (1.0)	71.63 (2.49)
*HME* (deg)	−14.03 (7.32)	52.92 (1.89)
*KME_sw* (deg)	−0.10 (6.25)	98.60 (2.11)
*KME_st* (deg)	0.09 (5.93)	0.00 (2.093)
*KMF_st* (deg)	41.63 (6.10)	62.15 (2.46)
*HMER_st* (deg)	5.92 (7.41)	61.43 (62.58)
*HMER_sw* (deg)	11.37 (7.83)	71.96 (3.32)

**Table 4:** Gait metric comparisons between CP (*noFES1*) and typically developing reference dataset (*TD*).

Case	1	2	3	4	5	6
*AGRF* (N/kg)	**	**	**	**	**	0.031
*TC_sw* (cm)	**	**	**	**	**	**
*HME* (deg)	**	N/A	**	N/A	**	**
*KME_sw* (deg)	N/A	N/A	**	N/A	N/A	**
*KME_st* (deg)	**	**	**	**	**	N/A
*KMF_st* (deg)	N/A	**	N/A	N/A	N/A	N/A
*HMER_st* (deg)	N/A	N/A	N/A	**	N/A	N/A
*HMER_sw* (deg)	N/A	**	N/A	N/A	N/A	N/A

N/A: not targeted by stimulation program, ** *p* < 0.001.

## Data Availability

The data presented in this study are available on request from the corresponding author.

## Supplementary Materials

The following are available online at https://www.mdpi.com/article/10.3390/s21134452/s1, Figure S1: Stimulation thresholding flow chart, Table S1: Eligibility Criteria.
