# Supplementary material for: Evaluation of Individualized Functional Electrical Stimulation-Induced Acute Changes during Walking: A Case Series in Children with Cerebral Palsy
Source: Sensors (Basel). 2021 Jun 29;21(13):4452. doi: 10.3390/s21134452 (PMC8271667; doi:10.3390/s21134452)
Supplement: Supplementary file 1 [file sensors-21-04452-s001.zip › sensors-1223115-supplementary.pdf]

Supplementary Materials:

Stimulation thresholding flow chart

The functional level stimulation settings were established for each muscle group included in the individualized stimulation program based on the goal associated with the position and joint (Figure S1). The stimulation thresholding flow chart (Figure S1) indicates: the general position the participant was evaluated in, the joint that was observed when thresholding, the muscle group that was targeted, a secondary position (if indicated), and the goal of the stimulus; which was used to determine the current amplitude and pulse duration. Stimulation thresholding was performed, for the muscle groups of interest, from the left to the right of the flow chart, in standing and followed by single leg standing, for the (1) hip, (2) knee, and (3) ankle on the right side followed by the left side.

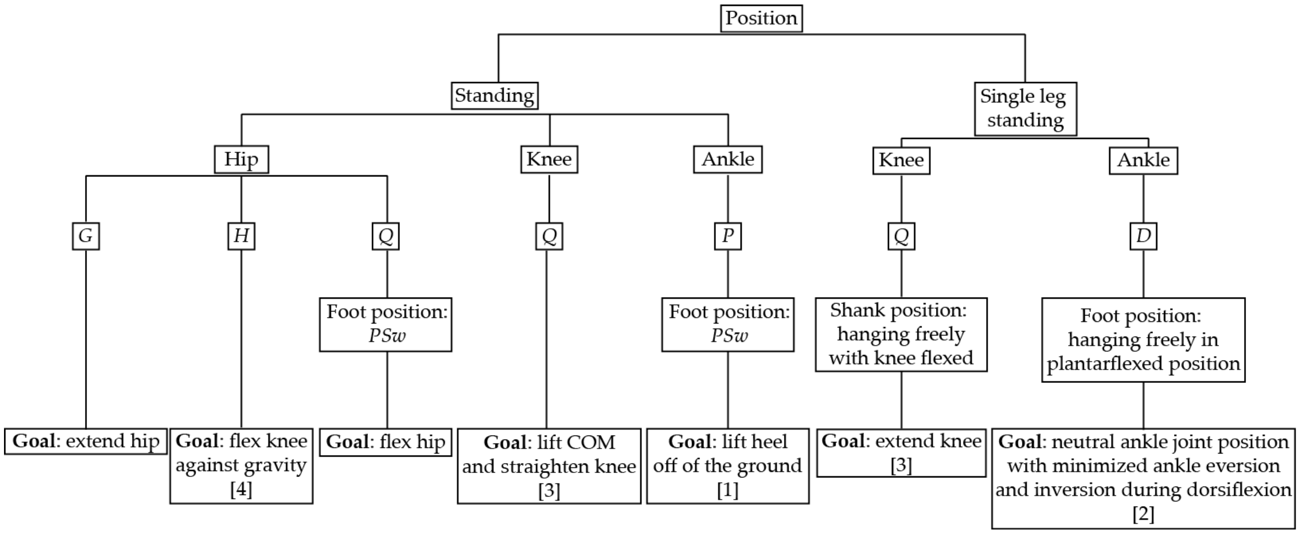

Figure S1. Stimulation thresholding flow chart. The flow chart indicates the position the lower extremity joints were tested in, the muscle groups associated with each joint, and the goal associated with the position/joint/muscle group. These were the criteria used to set the stimulation pulse duration. A secondary position was noted for some of the joints and muscle groups to position the limb correctly for specific phases of the gait cycle. P: plantarflexors [1], D: dorsiflexors [2], Q: quadriceps [3], H: hamstrings [4], G: gluteals, COM: center of mass.

For the quadriceps, three different stimulation thresholds were outlined in Figure S1, however flexion at the hip was unattainable with stimulation to the quadriceps when the foot was placed in pre-swing position for all participants. This finding was not surprising because the psoas major and iliacus muscles are too deep to be comfortably reached with transcutaneous electrodes. Although a portion of the electrode was on the distal rectus femoris, we did not try to preferentially recruit the rectus femoris separate from the remaining quadriceps. The values for the quadriceps associated with meeting the goals of (1) lifting the COM and straightening the knee, and (2) extending the knee were incorporated into the software for the associated gait phases (i.e. the pulse duration that creates a lift in the COM and straightens the knee was used during the mid-stance phase of gait).

Table S1. Eligibility Criteria.

Inclusion	Exclusion
Age 10-18	Diagnosis of athetoid or ataxic CP
The diagnosis of spastic diplegic CP	Significant scoliosis with primary curve > 40°
Classified as levels I-III on the Gross Motor Function Classification System [5]	Spinal fusions extending into the pelvis
Sufficient covering of the femoral head in the acetabulum (Reimers' hip migration percentage<40%)	Severe tactile hypersensitivity
Exhibit typical gait deviations characterized in CP (i.e. crouch, equinus, jump gait)	Joint instability or dislocation in the lower extremities
Achieve at least 0° of dorsiflexion during passive ROM in physical exam	Lower extremity surgery or fractures in the past year
Visuoperceptual skills and cognitive/ communication skills to follow multiple step commands for attending to exercise and data collection	Botulinum toxin injections in the LE muscles within the past 6 months
Seizure-free or well controlled seizures and no other neurological or musculoskeletal diagnoses such as dystonia, severe scoliosis, or hip instability	Implanted medical device contraindicated with the application of FES
Willingness to participate in testing and training sessions as prescribed by the study	Severe spasticity of the leg muscles (i.e. A score of 4 on the Modified Ashworth Scale)
Ability to communicate pain or discomfort with testing and training procedures	Uncharacteristic lower extremity joint pain during walking
Ability to obtain Parental/guardian permission and child assent	History of pulmonary disease limiting exercise tolerance (Asthma Control Test screen) or history of known cardiac disease (American Heart Association screen).
	Severely limited range of joint motion/ irreversible muscle contractures, i.e.> 10° knee flexion, >15° hip flexion contractures
	Pregnancy

References

1. Kesar, T.M.T.M.; Perumal, R.; Reisman, D.S.D.S.; Jancosko, A.; Rudolph, K.S.; Higginson, J.S.; Binder-Macleod, S.A. Functional electrical stimulation of ankle plantarflexor and dorsiflexor muscles: Effects on poststroke gait. *Stroke* **2009**, *40*, 3821–3827.
2. Kesar, T.T.M.; Perumal, R.; Jancosko, A.; Reisman, D.S.; Rudolph, K.S.; Higginson, J.S.; Binder-Macleod, S.A. Novel patterns of functional electrical stimulation have an immediate effect on dorsiflexor muscle function during gait for people poststroke. *Phys. Ther.* **2010**, *90*, 55–66.
3. van der Linden, M.L.; Hazlewood, M.E.; Hillman, S.J.; Robb, J.E. Functional Electrical Stimulation to the Dorsiflexors and Quadriceps in Children with Cerebral Palsy. *Pediatr. Phys. Ther.* **2008**, *20*, 23–29.
4. Stewart, C.; Postans, N.; Schwartz, M.H.; Rozumalski, A.; Roberts, A.P. An investigation of the action of the hamstring muscles during standing in crouch using functional electrical stimulation (FES). *Gait.Posture.* **2008**, *28*, 372–377.
5. Palisano, R.; Rosenbaum, P.; Bartlett, D.; Livingston, M. Content validity of the exapnded and revised Gross Motor Function Classification System. *Dev. Med. Child Neurol.* **2008**, *50*, 744–50.
